# The Emergence of Hierarchical Somatosensory Processing in Late Prematurity

**DOI:** 10.1093/cercor/bhz030

**Published:** 2019-03-07

**Authors:** K Whitehead, C Papadelis, M P Laudiano-Dray, J Meek, L Fabrizi

**Affiliations:** 1 Department of Neuroscience, Physiology and Pharmacology, University College London, London, UK; 2 Laboratory of Children’s Brain Dynamics, Fetal-Neonatal Neuroimaging and Developmental Science Center, Division of Newborn Medicine, Boston Children’s Hospital, Harvard Medical School, Boston, MA, USA; 3 Neonatal Unit, Elizabeth Garrett Anderson Wing, University College London Hospitals, London, UK

**Keywords:** brain development, EEG, ERP, mechanical stimulation, pre-term neonates

## Abstract

The somatosensory system has a hierarchical organization. Information processing increases in complexity from the contralateral primary sensory cortex to bilateral association cortices and this is represented by a sequence of somatosensory-evoked potentials recorded with scalp electroencephalographies. The mammalian somatosensory system matures over the early postnatal period in a rostro-caudal progression, but little is known about the development of hierarchical information processing in the human infant brain. To investigate the normal human development of the somatosensory hierarchy, we recorded potentials evoked by mechanical stimulation of hands and feet in 34 infants between 34 and 42 weeks corrected gestational age, with median postnatal age of 3 days. We show that the shortest latency potential was evoked for both hands and feet at all ages with a contralateral somatotopic source in the primary somatosensory cortex (SI). However, the longer latency responses, localized in SI and beyond, matured with age. They gradually emerged for the foot and, although always present for the hand, showed a shift from purely contralateral to bilateral hemispheric activation. These results demonstrate the rostro-caudal development of human somatosensory hierarchy and suggest that the development of its higher tiers is complete only just before the time of normal birth.

## Introduction

The processing of somatosensory information in the mammalian cortex has a hierarchical organization. Features of increasing complexity are encoded in an ascending system of connected cortical areas and functions to allow, for example, the localization and then conscious perception of the various qualities of touch (Felleman and Essen 1991; Iwamura 1998; Dijkerman and Haan 2007). Areas within primary somatosensory cortex (SI) sequentially encode the location and characteristics of contralateral somatosensory input, before other areas integrate information from the 2 body sides and integrate multiple sensory modalities, facilitating object recognition and motor planning (Kandel et al. 2000). Early somatosensation may contribute to survival behaviors such as feeding, rudimentary motor skills like grasping, and the beneficial effects which parenting provides (Colson et al. 2008; Hackman et al. 2010; Molina et al. 2015).

In rodents, functional somatosensory architecture develops over the first few postnatal weeks (McVea et al. 2012), which correspond to the last trimester of gestation in humans (Clancy et al. 2007) and follows a rostro-caudal developmental gradient. This is clear from the sequential organization of SI: (i) neurons in SI initially respond almost exclusively to whiskers stimulation, while responses to forelimb appear later and are finally followed by responses to hindlimb stimulation (McCandlish et al. 1993; Seelke et al. 2012); (ii) amputation of the forelimb at P0 results in the corresponding SI representation to respond to hindlimb, but not whiskers, stimulation suggesting that inputs from the facial area have already matured at birth (Pluto et al. 2003). In addition, complex sensorimotor interactions such as placing (i.e., lifting a paw and placing the sole flat on a support platform) in response to a gentle touch develops for the forelimb before the hindlimb (Donatelle 1977). In line with these findings in animal models, upper limb grasp is more common than lower limb grasp in full-term neonates (Colson et al. 2008). However, little is known about when the different levels of the somatosensory processing pathway mature in humans, or whether this maturation occurs first for the upper limbs.

The connections which allow the flow of tactile information along the somatosensory processing pathway structurally develop and refine across the third trimester and perinatal period. Cortical Layer IV begins to differentiate between 20 and 26 weeks of gestation (Burkhalter et al. 1993; Rees et al. 2010) with early thalamo-cortical contacts, synaptogenesis and vertical inter-layer connections occurring from 24 to 26 weeks (Flower 1985; Burkhalter et al. 1993; Volpe 2009). However, synaptogenesis of thalamo-cortical and cortico-cortical connections is most pronounced from 28 weeks until full-term age, in line with extensive dendritic development (Flower 1985). The entry of callosal fibers into the cortex from 33 weeks (Volpe 2009), disappearance of the somatosensory subplate from 36 weeks (Kostovic and Rakic 1990), dense intra-layer horizontal cortical connections by 37 weeks (Burkhalter et al. 1993), and a peak in axon growth within the parietal white matter at 38–42 weeks (Haynes et al. 2005) suggests that the inter- and intra-hemispheric cortico-cortical circuits necessary for higher-order somatosensory functioning may mature within the late pre-term and perinatal period. Indeed, functional magnetic resonance imaging (f)MRI experiments indicate that the equivalent of the last trimester of gestation is characterized by more spatially complex functional somatosensory responses with increasing integration of the ipsilateral hemisphere and association cortices (Allievi et al. 2016).

Somatosensory processing can be investigated with high temporal resolution by recording scalp electroencephalography (EEG) and analyzing the multiple somatosensory-evoked potentials (SEPs) arising in response to stimulation. Indeed, in adults, SEPs have been linked to different levels of the processing hierarchy of tactile information: early SEPs, consistent with a source in SI (Allison et al. 1992), are recorded in adults even if stimuli do not elicit conscious perception, whereas later SEPs from 80 to 500 ms, likely to be generated beyond SI (Frot and Mauguière 1999; Hoechstetter et al. 2001), are only recorded if the stimulus has entered awareness (Libet et al. 1967; Kitazawa 2002).

The maturation of higher-level somatosensory processing in the developing pre-term brain is poorly understood because the majority of studies have focussed only on the primary afferent volley. At 29–33 weeks, a single high amplitude negative response can be elicited by somatosensory stimulation, of maximal amplitude at the contralateral central region following hand stimulation and at the midline central region after foot stimulation (Hrbek et al. 1973; Milh et al. 2007; Vanhatalo et al. 2009; Whitehead et al. 2016, 2018) while by full-term age the somatosensory response comprises a sequence of positive and negative SEPs, as in adults (Karniski et al. 1992; Fabrizi et al. 2011). The first of these events in response to hand stimulation is a negative-positive complex (N1–P1) (Desmedt and Manil 1970; Hrbek et al. 1973; Laget et al. 1976; Karniski et al. 1992; Taylor et al. 1996) over the contralateral central area occurring between 30 and 100 ms, or a positive deflection (P1, 37–50 ms) over the midline central area following stimulation of the foot (Vaughan 1975; Georgesco et al. 1982; Gilmore et al. 1987; White and Cooke 1989; Minami et al. 1996; Pike et al. 1997). The somatotopic organization of the electric and magnetic field of these early potentials is that of a forward pointing dipole consistent with activity in Brodmann Area (BA) 3b of the SI representation of the stimulated limb, indicating the arrival of the peripheral afferent volley (Minami et al. 1996; Pike et al. 1997; Pihko et al. 2004; Lauronen et al. 2006).

Much less is known about longer-latency potentials, which are considered to reflect higher-order processing levels further along the hierarchical tree (Nevalainen et al. 2014; Saby et al. 2016). At full-term, stimulation of the hands and feet elicits a second negative deflection (N2) at 150 ms following the early N1 and/or P1, and, less consistently reported, a second positive peak (P2) at 240 ms and a third negative peak (N3) at 450 ms (hands: Desmedt and Manil 1970; Hrbek et al. 1973; Laget et al. 1976; Karniski et al. 1992; Taylor et al. 1996; Pihko et al. 2004; Nevalainen et al. 2015; Maitre et al. 2017; Donadio et al. 2018); feet: (Cindro et al., 1985; Minami et al. 1996; Pike et al. 1997; Slater et al. 2010; Fabrizi et al. 2011; Donadio et al. 2018). These potentials emerge over the equivalent of the last trimester of gestation (Hrbek et al. 1973; Fabrizi et al. 2011), but when each tier of the hierarchical chain is established is not known.

We hypothesized that the inter- and intra-hemispheric cortical changes that underpin somatosensory processing could be functionally reflected in the emergence of specific SEPs and changes in their source in humans. To address this, we recorded SEPs following tactile stimulation of all 4 limbs in late pre-term and full-term neonates with a corrected gestational age (CGA) of 34–42 weeks where CGA is defined as gestational age (GA) at birth + postnatal age. To ensure that our data reflect intrinsic somatosensory maturation, and are not affected by experience, our cohort has a median postnatal age of just 3 days. We then mapped the emergence and topographical and source localization changes of each potential across CGA.

## Materials and Methods

### Subjects

34 infants evenly spread between 34 + 5–42 + 5 CGA (weeks + days) were recruited for this study from the postnatal ward and special care baby unit at the Elizabeth Garrett Anderson wing of University College London Hospitals between September 2015 and July 2016 (Table 1). No neonates were acutely unwell, receiving neuroactive medication or receiving respiratory support at the time of study. Infants were neurologically normal both at the time of study and at the date of discharge based on review of medical notes and the discharge summary. No subjects had congenital abnormalities except for a single neonate with a cleft lip. Cranial ultrasound scans were reported as normal when subjects were referred for one (*n* = 6, including the baby with a cleft lip). All EEGs were assessed as normal for CGA by a clinical neurophysiologist (KW) according to Tsuchida et al. (2013): developmental features included alternating patterns and frequent delta brushes in the youngest infants and continuous multi-frequency activity, with no delta brushes, in the oldest infants (Supplementary Fig. S1).

**Table 1:** Demographics of the sample population divided into 4 age groups

	Total	Pre-term	Early-term	Full-term	Late-term
No. of neonates	34	9	9	8	8
Median (range) CGA at time of study (weeks+days)	38 + 1	35 + 4	37 + 4	40 + 0	41 + 5
	(34 + 5–42 + 5)	(34 + 5–36 + 4)	(37 + 0–38 + 1)	(39 + 2–40 + 3)	(41 + 0–42 + 5)
Median (range) GA at birth (weeks+days)	37 + 6	35 + 2	37 + 0	39 + 5	41 + 1
	(34 + 2–41 + 4)	(34 + 2–36 + 0)	(35 + 5–38 + 0)	(38 + 6–40 + 2)	(40 + 2–41 + 4)
Median (range) postnatal age at study (days)	3 (1–11)	4 (2–5)	3 (1–11)	1 (1–3)	5 (1–11)
Median (range) birth weight (g)	2810	2270	2680	3200	3605
	(1780–3968)	(1780–2910)	(2280–3170)	(2250–3630)	(2790–3968)
% Males	44	33.3	33.3	37.5	75.0
No. multiple gestation neonates	3	1	2	0	0

CGA indicates corrected gestational age; GA indicates gestational age; SD indicates standard deviation.

Ethical approval was obtained from the NHS Research Ethics Committee, and informed written parental consent was obtained prior to each study. The study conformed to the standards set by the Declaration of Helsinki guidelines and was well tolerated: 28/30 of the neonates who were asleep at study onset slept through the whole protocol.

Vigilance state prior to stimulation of each limb was categorized according to EEG and respiratory criteria as wakefulness or active sleep in 72/113 and quiet sleep in 41/113 with no significant difference according to CGA (binary logistic regression *P* = 0.124), or which of the 4 limbs was stimulated (Pearson Chi-square *P* = 0.433).

### EEG Recording

Eighteen recording electrodes (disposable Ag/AgCl cup electrodes) were positioned according to the modified international 10/10 electrode placement system, with high-density central–parietal and temporal coverage, at F7, F8, F3, F4, Cz, CPz, C3, C4, CP3, CP4, T7, T8, P7, P8, TP9, TP10, O1, and O2. A reduced number of electrodes were applied if the infant became unsettled during set-up (median 18; 30/33 infants had ≥16 electrodes). The reference electrode was placed at Fz (Pike et al. 1997; Tombini et al. 2009; Vanhatalo et al. 2009; Trollmann et al. 2010) and the ground electrode was placed at FC1/2. Target impedance of electrodes was <10 kΩ (André et al. 2010). A single lead I ECG was recorded from both shoulders. Respiratory movement for sleep staging was monitored with an abdominal transducer. EEG was recorded with a direct current (DC)-coupled amplifier from DC-800 Hz using the Neuroscan (Scan 4.3) SynAmps2 EEG/EP recording system. Signals were digitized with a sampling rate of 2 kHz and a resolution of 24 bit.

### Tactile Stimulation

Mechanical taps were delivered by KW to the lateral edge of the infants’ palms and heels using a hand-held tendon hammer with a 15-mm^2^ contact surface (Supplementary Videos 1 and 2). The hammer had a piezo-electric transducer that allowed to measure the force applied at each tap, and to record the precise timing of the stimulation on the EEG recording (Worley et al. 2012). A train of maximum 48 somatosensory stimuli was delivered to each limb. The interstimulus interval was large, variable, and self-paced by the experimenter (8–15 s) as shorter intervals could attenuate long latency SEPs (Desmedt and Manil 1970; Gibson et al. 1992; Nevalainen et al. 2015). In case the infant moved, the tap was delayed for several seconds to avoid potential modulation of the somatosensory response by the movement (Saby et al. 2016) and to allow movement artifacts to resolve. The sequence in which the limbs were stimulated varied across subjects. In 7 neonates, it was not possible to stimulate one of the 2 hands because of the presence of a cannula, and a reduced amount of stimuli were delivered if the baby became unsettled. This resulted in a total of 113 stimulation trains (i.e., stimulated limbs) of 6–48 stimuli (mean ± SD: 19 ± 8.1) with a mean (±SD) force of 267 (±71) mN.

### Data Pre-processing

Data pre-processing was carried out using EEGLAB v.13 (Swartz Center for Computational Neuroscience). Data were downsampled to 512 Hz, bandpass filtered at 1.5–40 Hz (second-order Butterworth filter) with a 50-Hz notch filter (fourth-order Butterworth filter) and then epoched from −400 until +1300 ms around the stimulus. Although high-pass filtering can distort slow components of the somatosensory response (Pihko and Lauronen 2004), it is widely used to detect short-duration potentials characteristic of mature somatosensory responses (George and Taylor 1991). 23 epochs from 18 datasets containing movement artifact were completely discarded, and 16 datasets were de-noised using independent component analysis (independent components representing (i) transient electrode “pop”, (ii) sinusoidal electrical interference, (iii) rapid eye movements, and (iv) ECG breakthrough were removed) (Onton and Makeig 2006). This resulted in a total of 2104 epochs analyzed. Bad channels (poor contact with the scalp) were removed and then estimated with spherical interpolation as implemented in EEGLAB. All EEG epochs were re-referenced to common average (retrieving the reference channel Fz), baseline corrected by subtracting the mean baseline signal (−200 to 0 ms) and averaged across repetitions (i.e., each subject was characterized by a single average response per limb stimulated).

### Analysis of Somatosensory Response

#### Grand Average Analysis

We first identified the SEPs present following the stimulation of each limb. The grand average of the EEG response and its global field power (GFP) to left hand (LH), right hand (RH), left foot (LF), and right foot (RF) stimulation across all subjects was calculated. SEPs latencies were identified as local GFP maxima using the MATLAB function *findpeaks.m*. A local GFP peak was defined as a data sample that is larger than its 2 neighboring samples, has an amplitude of more than 1 μV and a prominence of more than 0.15 μV. The prominence of a peak indicates the extent by which a peak stands out in relation to other neighboring peaks (for a full definition of prominence refer to the MATLAB documentation for the function *findpeaks.m*).

#### SEPs Emergence Analysis

We then investigated the changes in SEPs occurrence with CGA. Individual SEPs presence was established at subject level with a 2 steps approach: (i) definition of a *spatio-temporal region of interest (ROI)* from the grand averages and (ii) assessment of individual peaks.

**Figure 1.**
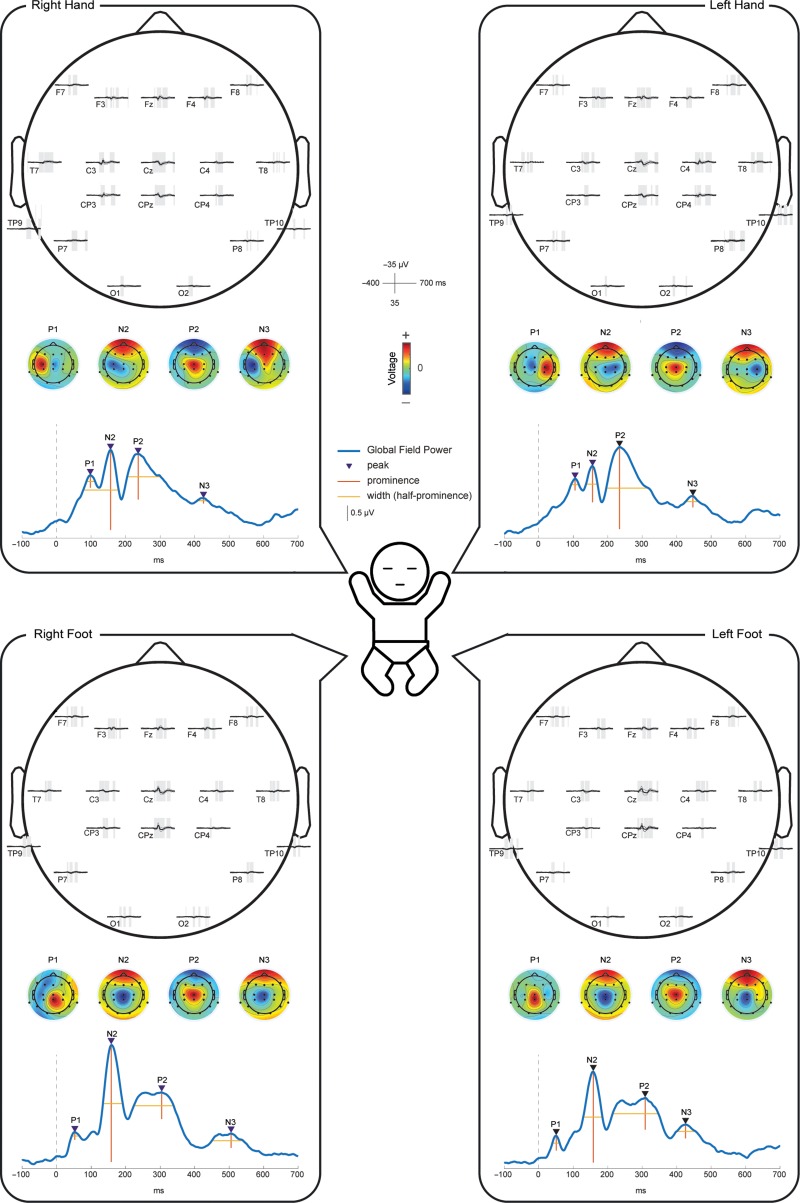
Grand average of the EEG responses following mechanical stimulation of all 4 limbs. Within each panel we displayed: (i) the grand average of the recordings at each electrode, where shading represent significant deflections (*P* < 0.05) from baseline; (ii) the global field power (GFP) of the grand average with marked local maxima representing the SEPs; and (iii) topographies of each SEP (normalized by GFP).

We first defined the *spatio-temporal ROI* within which individual peaks had to fall to be considered present. To define the *spatial ROI*, we plotted the topographies of the grand averages at the latencies of the local GFP peaks (4 peaks × 4 limbs, Fig. 1) and marked the equipotential line at half maximum of the largest peak at each latency (Supplementary Fig. S2). This is equivalent to determining the 2D Full-Width at Half-Maximum of the peaks. The spatial ROI was defined as the union of all the equipotential lines and encompassed the pericentral electrodes (C3, C4, CP3, CP4, Cz, CPz) (Supplementary Fig. S2). The *temporal ROI* was defined as the time interval in which the recording from at least one of the electrodes within the spatial ROI significantly deflected from baseline. A significant deflection (*P* < 0.05) was determined with a point-by-point *t*-test comparing each time point following stimulation (standard deviation (SD) calculated across subjects) to baseline (SD calculated across subjects and time).

Individual SEPs were then identified as local temporal and spatial maxima/minima occurring within the spatio-temporal ROI. Peaks potentially representing SEPs were first identified from the recordings at the pericentral electrodes using the MATLAB function *findpeaks.m.* These were data samples that were larger/smaller than their 2 neighboring samples, had a prominence of more than 2 μV, width at half-prominence of more than 14 ms and occurred within the temporal ROI (Supplementary Fig. S3). If more data points satisfied these criteria the latency of that closest to the grand average SEP was selected. If no data point satisfied these criteria the SEP was considered absent. If a peak potentially representing an SEP was present, its topography was assessed against the spatial ROI. Spatial maxima/minima at the latencies of the selected peaks were identified using the MATLAB functions *imregionalmax.m* and *imregionalmin.m* (Supplementary Fig. S3). If the spatial maxima/minima fell within the boundary of the spatial ROI the SEP was finally considered present. Changes in occurrence (and amplitude) of each potential according to CGA were then assessed using a logistic (and linear) regression model for hands and feet separately. In this analysis, a significant positive regression coefficient represents an increase in the occurrence of a potential with age. Throughout, the 95% confidence interval was calculated using parametric bootstrapping.

To provide a visual representation of the developmental changes in the somatosensory response waveform, we generated average traces for the contralateral and midline pericentral electrodes for each of 4 age groups (pre-term, early-term, full-term, and late-term, Table 1).

#### SEPs Topography Development Analysis

We then investigated changes in the SEPs topographies with CGA using global dissimilarity (DISS), which quantifies differences between 2 electric fields, sampled at the scalp, independently of their strength (Murray et al. 2008; Tzovara et al. 2012). We first calculated the mean topographies of each SEP for the oldest infants (CGA ≥ 42 weeks) by averaging the topographies (normalized by GFP) of the peaks classified as present (Figs 4 and 5 bottom row). We then compared the topographies (normalized by GFP) of each individual with these references by calculating DISS. This index ranges between 0 and 2, with 0 meaning that topographies are identical and 2 meaning that topographies are inverted. A topography was considered “similar” to the reference topography if DISS was lower than the median of all the DISS calculated (which was 0.819). Changes in occurrence of topographies “similar” to those of the oldest infants according to CGA were then assessed using a logistic regression model for hands and feet separately. The datasets used to calculate the reference topographies were excluded from this regression to not bias the results. In this analysis, a significant positive regression coefficient represents an increase in the occurrence of topographies “similar” to that of the oldest infants with age.

To explain changes in DISS, topographies were then classified into contralateral (*x* < −25°), midline (−25°< *x* < 25°) and ipsilateral (*x* > 25°) according to the angular distance of the main peak from the midline. Changes in occurrence of peaks with midline topography were then assessed using a logistic regression model for hands and feet separately. In this analysis, a significant positive regression coefficient represents an increase in the occurrence of peaks with midline topography with CGA.

To provide a visual representation of the developmental changes in the SEPs topographies, we generated average scalp maps for each of the 4 age groups (Table 1). Average scalp maps were obtained averaging data normalized by GFP from subjects for whom the SEPs were present.

#### Source Localization Analysis

We then localized the cerebral generators of the SEPs for each of the 4 age groups (Table 1). To estimate the source of activity from the scalp SEPs, we need to consider 2 distinct modeling problems: (i) the forward model (or head model) that represents the electromagnetic properties of the head and of the sensor array, and (ii) the inverse problem that estimates the brain sources which produced the scalp EEG data. Because cerebral anatomy changes rapidly over the developmental period considered in this study, we created an age-specific realistic head model for each of the 4 age groups (Routier et al. 2017). To do that, we used a 3-layer (scalp, 0.33 S/m; skull, 0.0042 S/m, and brain, 0.33 S/m) boundary element method (BEM) model of age-matched MRI templates (35 weeks CGA for the pre-term, 37 weeks for the early-term, 39 weeks for the full-term, and 41 weeks for the late-term group) derived from the Neonatal Brain Atlas (Serag et al. 2012). The reconstruction of the BEM models was performed using the OpenMEEG software (Kybic et al. 2005; Gramfort et al. 2010). Each layer consisted of 1082 vertices. A grid of points that sampled the full brain volume (volume points) was generated using an adaptive integration method, which is available in *Brainstorm* (Tadel et al. 2011). The full brain volume was used as source space. Anatomical landmarks (nasion, right, and left ears) were manually defined on the MRI images and used for EEG electrodes co-registration. The relative position of the EEG electrodes was taken from the MNI coordinates available in *Brainstorm* (Tadel et al. 2011).

We then solved the inverse problem with the equivalent current dipole (ECD) method for each subject for whom the SEPs were classified as present using the age-appropriate head model. ECD assumes that one focal source, described by an infinitesimally small line element (Hämäläinen et al. 1993), generates the observed scalp electrical activity and has been extensively used for the localization of SI activity in both neonates (Pihko et al. 2004; Nevalainen et al. 2015) and adults (Inui et al. 2004; Papadelis et al. 2011, 2012). The location, orientation, and moment of the dipole was estimated for each infant at the latency of each SEP using a source scanning method implemented in Brainstorm (Tadel et al. 2011). This method searches iteratively for the dipole explaining best the recordings without any a priori definition of the initialization point. Unconstrained source analysis was performed in the volume space for each infant. For each dipole, we calculated the goodness-of-fit (GOF) that indicates the percentage of the data that can be explained by the model. Only dipoles with a GOF > 80% were considered for group analysis. High values of GOF indicate that the EEG signal is dominated by the contribution from a single focal source. Other possibly simultaneously active sources are either uncorrelated with the stimulus and thus reduced by averaging or have been attenuated by filtering (Papadelis et al. 2011). For each SEP, ECDs were classified as in-cluster or scattered for each of the 4 age groups, depending on their spatial contiguity. A cluster was defined as 5 or more dipoles located within a 20-mm distance for hands stimulation or 25-mm distance for feet stimulation. The ECD localization findings were superimposed on the age-specific template MRI. For each SEP, only ECD solutions which were in-cluster were regarded as reliable; for these solutions, the mean dipole was also estimated (ECD having as location, orientation, and moment the mean values of all dipoles).

## Results

### Mechanical Stimulation of the Limbs Evokes a Sequence of 4 SEPs

Mechanical stimulation of the LH, RH, LF, and RF consistently evoked a sequence of 4 SEPs: P1, N2, P2, and N3 (Fig. 1). The latencies of the peaks were at approximately P100-N150-P230-N440 for the hands and at P50-N160-P300-N450 for the feet.

### The Short-Latency P1 Potential is Already Established from 34 Weeks CGA

**Figure 2.**
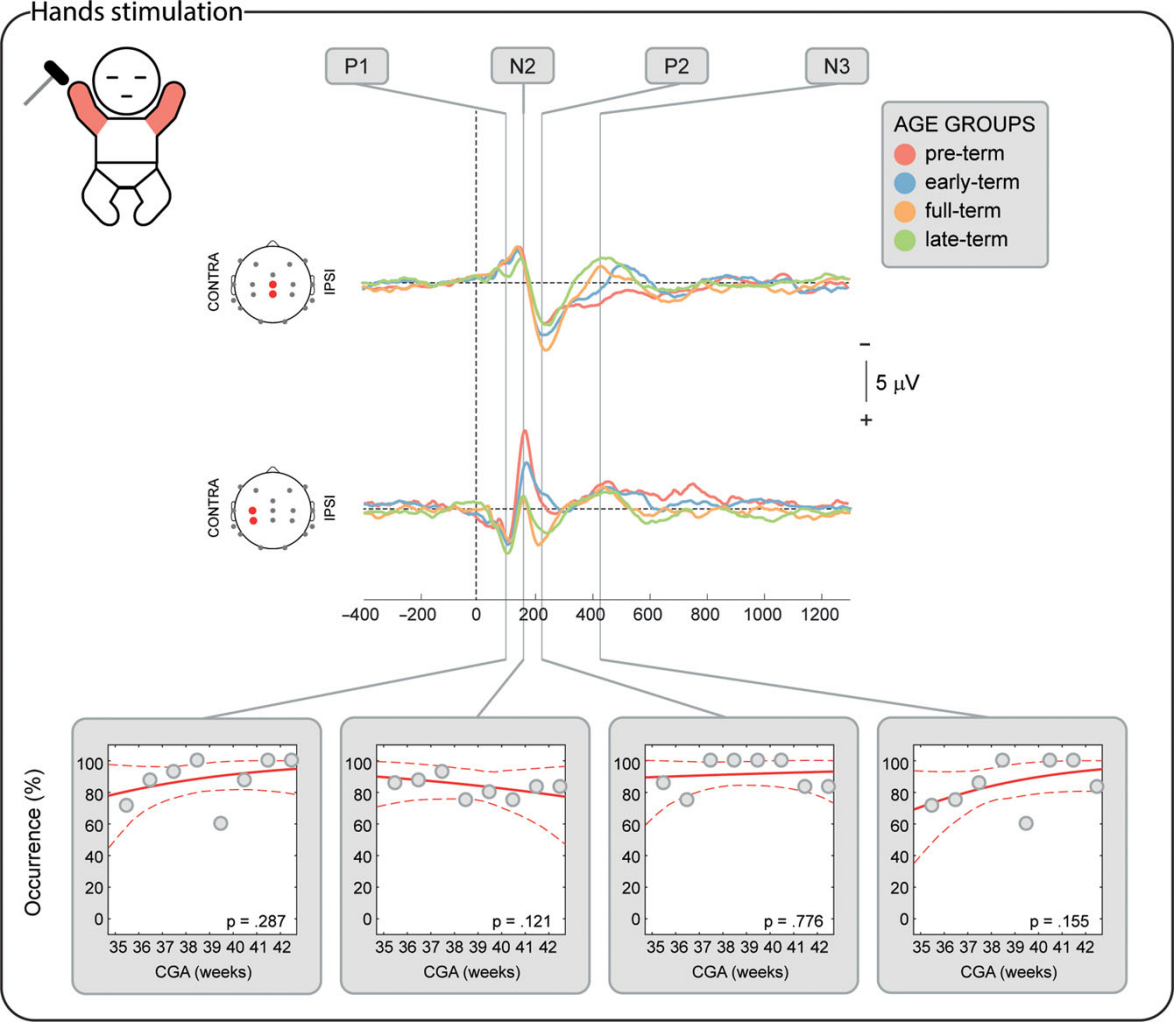
SEPs occurrence in response to the stimulation of both hands according to corrected gestational age (CGA) at time of study. Upper panel: illustrative mean response recorded at the midline and contralateral pericentral electrodes in 4 age groups (pre-term, early-term, full-term, and late-term) (Table 1). Bottom panels: occurrence of each potential in respect to CGA and significance of the correlation. Gray dots represent mean occurrence in 1-week windows (calculated only for illustrative purposes), the red solid line is the logistic regression curve and the dashed red lines delimit the 95% confidence interval.

**Figure 3.**
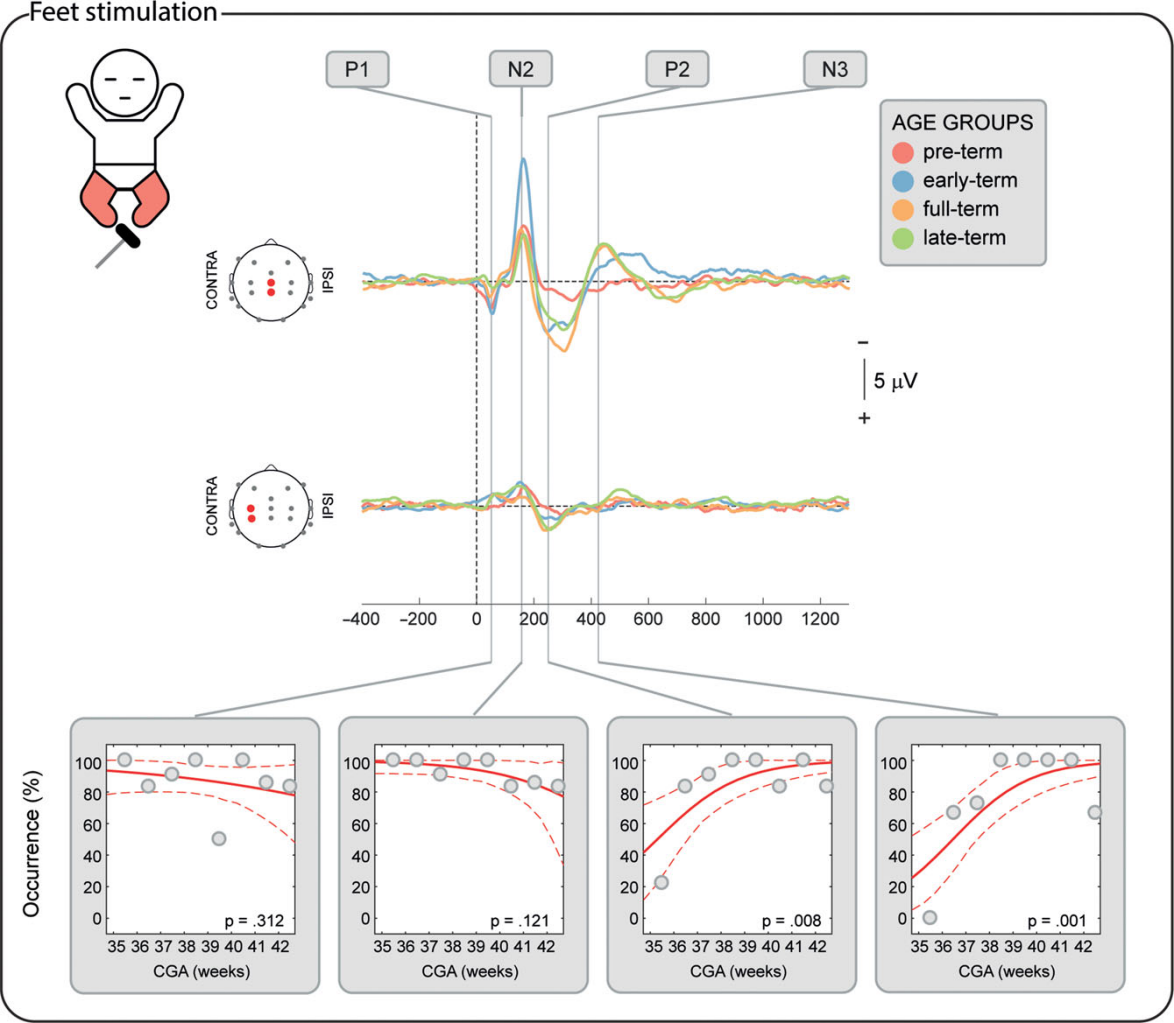
SEPs occurrence in response to the stimulation of both feet according to corrected gestational age (CGA) at time of study. Upper panel: illustrative mean response recorded at the midline and contralateral pericentral electrodes in 4 age groups (pre-term, early-term, full-term, and late-term) (Table 1). Bottom panels: occurrence of each potential in respect to CGA and significance of the correlation. Gray dots represent mean occurrence in 1-week windows (calculated only for illustrative purposes), the red solid line is the logistic regression curve and the dashed red lines delimit the 95% confidence interval.

**Figure 4.**
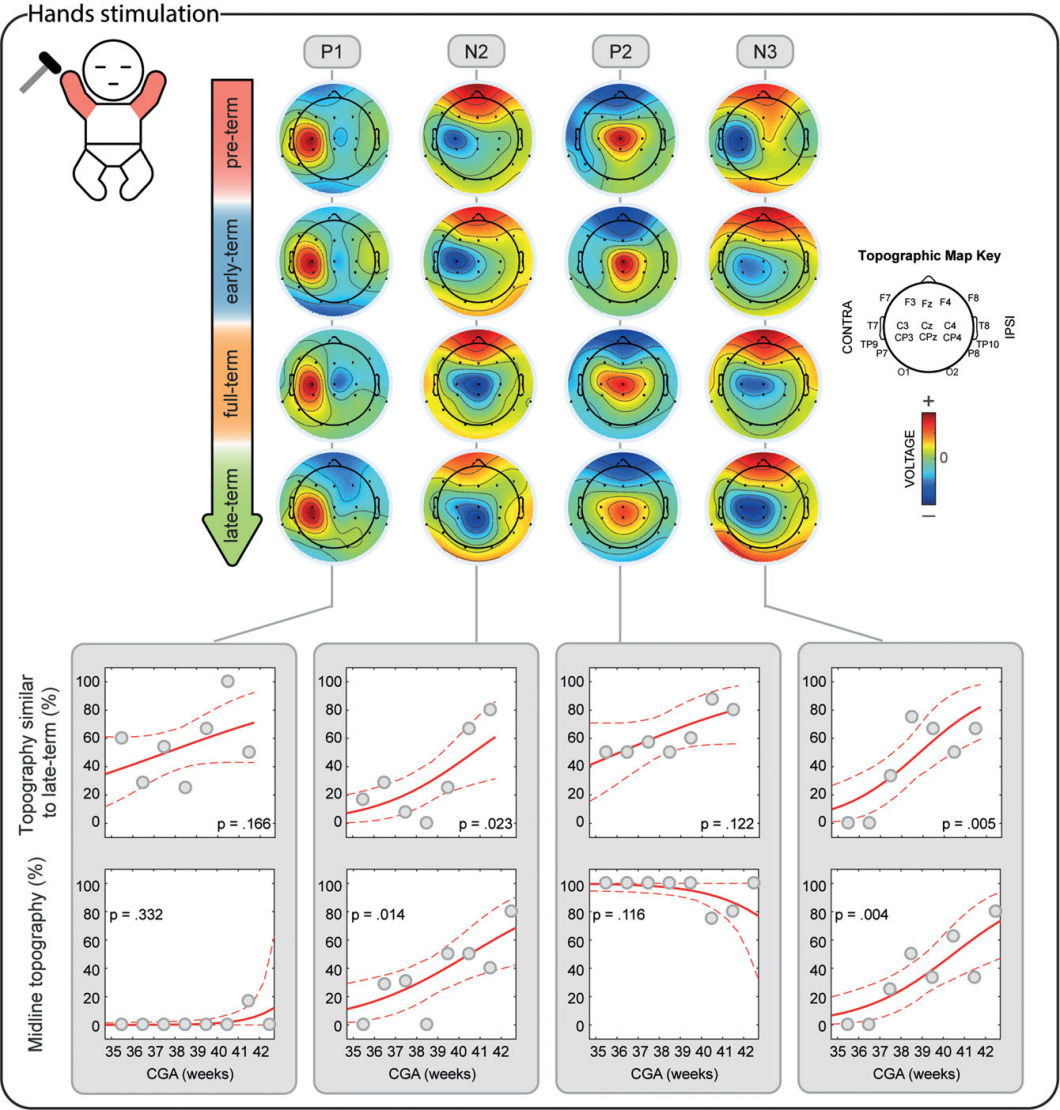
Development of the topographical distribution of the SEPs in response to the stimulation of both hands. Upper panel: illustrative mean topographical distribution within 4 age groups (pre-term, early-term, full-term, and late-term). The color scale is normalized to the maximum absolute value within each map. Bottom panels: occurrence of topographies “similar” to the topography at CGA ≥ 42 weeks and occurrence of midline topographies in respect to CGA at time of study. Gray dots represent mean occurrence of “similar” (upper graphs) or midline topographies (lower graphs) in 1-week windows (calculated only for illustrative purposes), the red solid line is the logistic regression curve and the dashed red lines delimit the 95% confidence interval.

**Figure 5.**
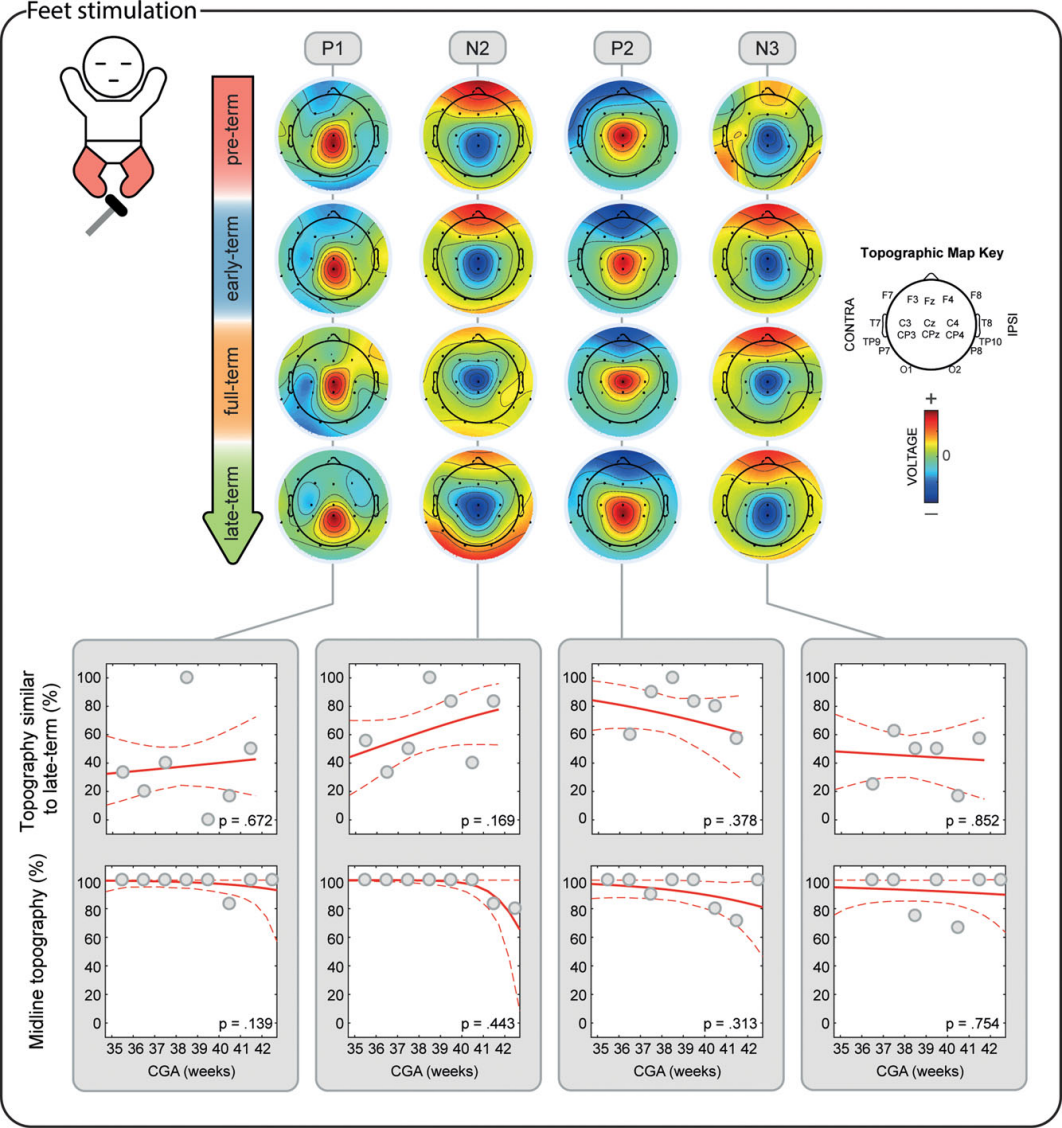
Development of the topographical distribution of the SEPs in response to the stimulation of both feet. Upper panel: illustrative mean topographical distribution within 4 age groups (pre-term, early-term, full-term, and late-term). The color scale is normalized to the maximum absolute value within each map. Bottom panels: occurrence of topographies “similar” to the topography at CGA ≥ 42 weeks and occurrence of midline topographies in respect to CGA at time of study. Gray dots represent mean occurrence of “similar” (upper graphs) or midline topographies (lower graphs) in 1-week windows (calculated only for illustrative purposes), the red solid line is the logistic regression curve and the dashed red lines delimit the 95% confidence interval.

**Figure 6.**
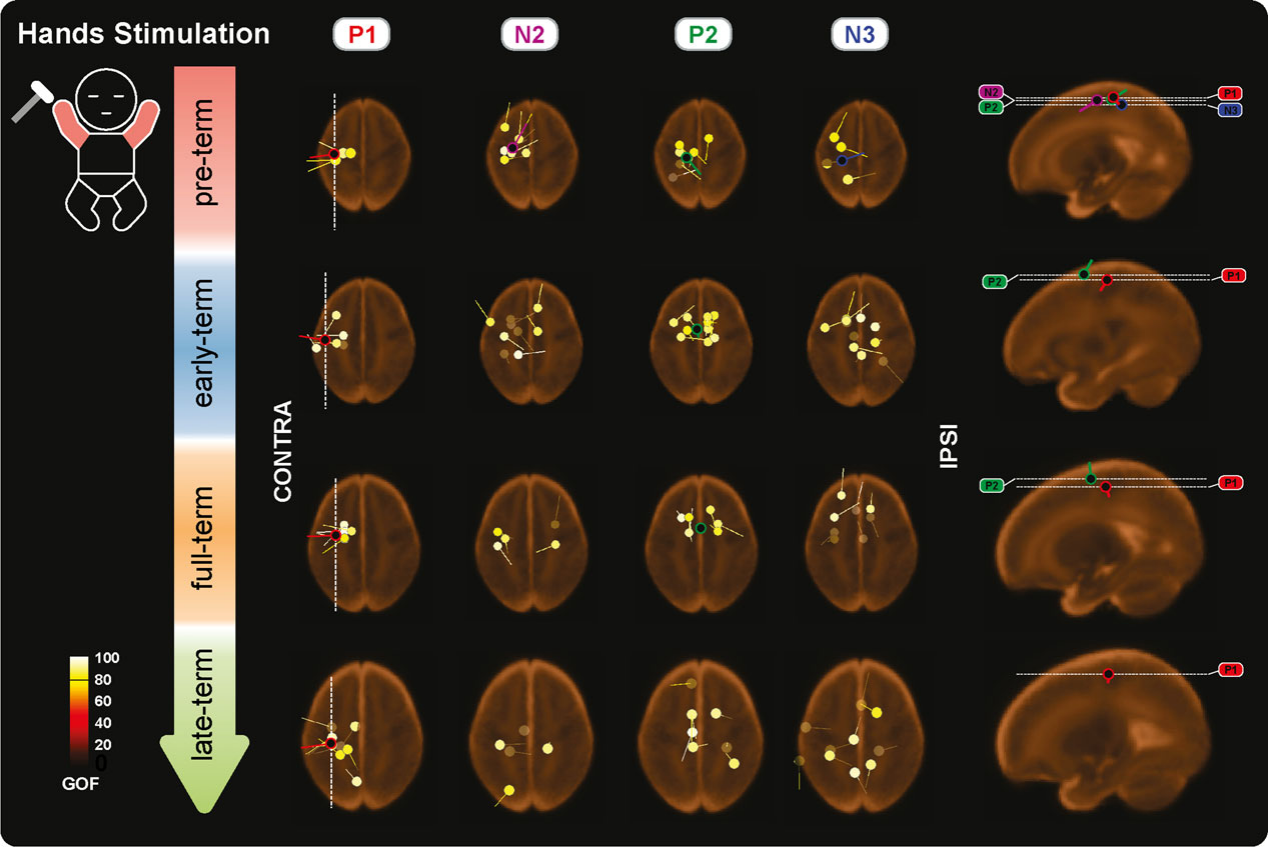
Individual and mean equivalent current dipoles (ECDs) locations for P1, N2, P2, and N3 superimposed on age-specific neonatal MRI templates for stimulation of both hands. The individual ECDs are color coded based on their goodness-of-fit (GOF) and displayed separately for each SEP (P1, N2, P2, and N3) and age group (pre-term, early-term, full-term, and late-term). Only individual ECDs with GOF > 80% and mean dipoles for in-cluster localization solutions (>5 dipoles located within a 20-mm distance) are displayed. ECDs are projected on the axial slice passing through the center of the ECDs distribution. The dorsoventral positions of the axial slices are marked (white dashed lines) on the sagittal view on the right together with the mean dipoles. The mediolateral position of the sagittal slices is marked on the axial slices for P1 (white dashed lines).

**Figure 7.**
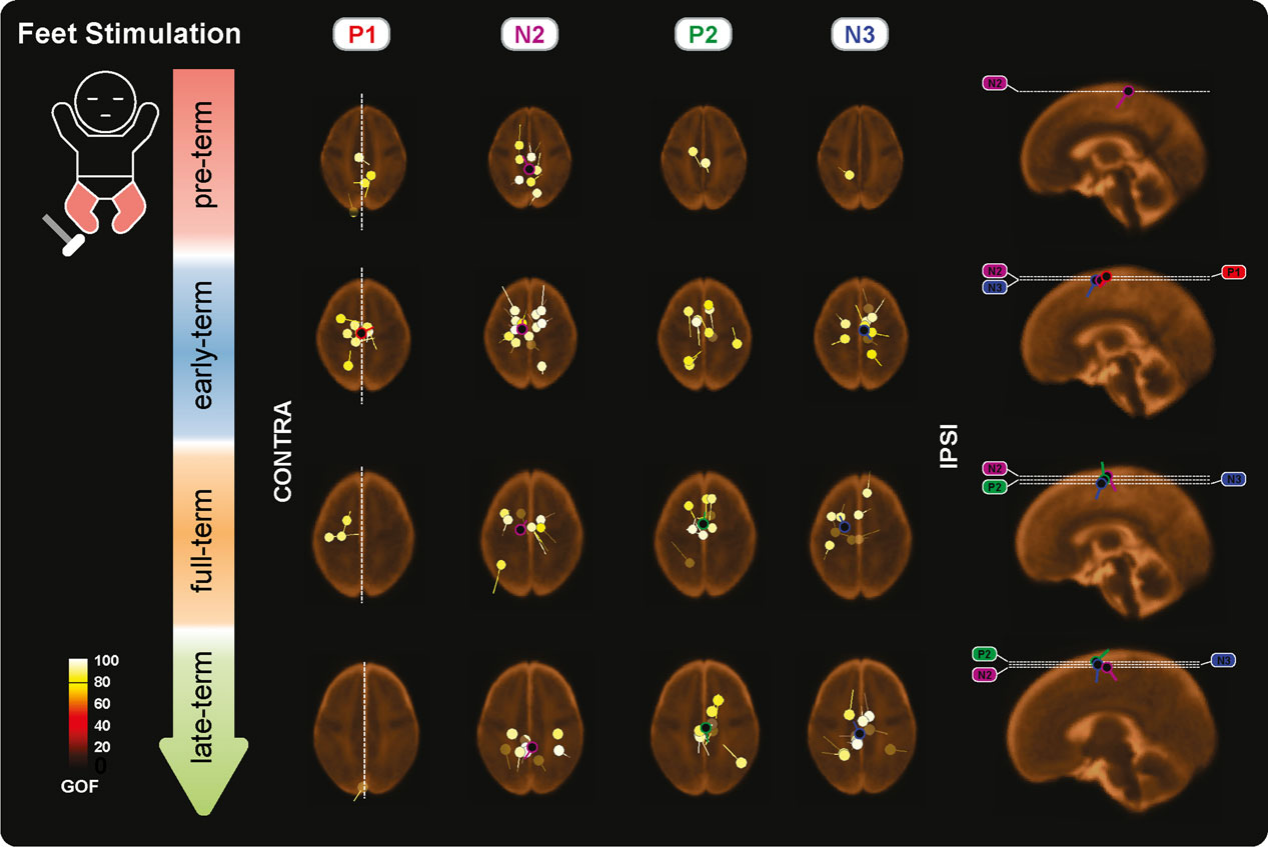
Individual and mean equivalent current dipoles (ECDs) locations for P1, N2, P2, and N3 superimposed on age-specific neonatal MRI templates for stimulation of both feet. The individual ECDs are color coded based on their goodness-of-fit (GOF) and displayed separately for each SEP (P1, N2, P2, and N3) and age group (pre-term, early-term, full-term, and late-term). Only individual ECDs with GOF > 80% and mean dipoles for in-cluster localization solutions (>5 dipoles located within a 25-mm distance) are displayed. ECDs are projected on the axial slice passing through the center of the ECDs distribution. The dorsoventral positions of the axial slices are marked (white dashed lines) on the sagittal view on the right together with the mean dipoles. The mediolateral position of the sagittal slices is marked on the axial slices for P1 (white dashed lines).

P1 was recorded in 87.9% of the test occasions following hand stimulation and 87.3% following foot stimulation independently of the CGA of the infants (hands: *P* = 0.287; feet: *P* = 0.312, Figs 2–3) with a stable amplitude (hands (2.4 ± 0.6 μV [mean ± SD]): *P* = 0.839; feet (2.3 ± 0.7 μV): *P* = 0.312). When recorded, P1 had a stable topography (DISS vs. CGA, hands: *P* = 0.166; feet: *P* = 0.672, Figs 4–5) which was maximal contralaterally 98.0% of the times with a contralateral pericentral source (Fig. 6) for the hands and at the midline 97.9% of the times (with medial pericentral source at early-term, Fig. 7) for the feet independently of CGA (hands: *P* = 0.332; feet *P* = 0.139, Figs 4–5).

### The Long-Latency Potentials Mature Over the Late Pre-term and Perinatal Period

N2 was recorded in 84.5% of the test occasions following hand stimulation and 92.7% following foot stimulation independently of CGA (hands: *P* = 0.445; feet: *P* = 0.121, Figs 2–3). The amplitude following hand stimulation decreased from 2.6 μV before 36 weeks to 2.3 μV after 41 weeks (*P* = 0.018), but remained stable following foot stimulation (*P* = 0.633). The N2 topography following hand stimulation changed with age (DISS vs. CGA, *P* = 0.023, Fig. 4) with a shift from contralateral (100% before 36 weeks CGA with a contralateral pericentral source, Fig. 6) to midline (*P* = 0.014, no reliable source localization, Figs 4 and 6). N2 following foot stimulation had a stable topography (DISS vs. CGA, *P* = 0.169, Fig. 5) which was maximal at the midline 96.1% of the times independently of the CGA (*P* = 0.443) with a medial pericentral source (Fig. 7).

P2 was recorded in 91.4% of the hand stimulations independently of CGA (*P* = 0.776), but in only 22.2% of the foot stimulations before 36 weeks CGA and increased in occurrence with CGA (*P* = 0.008, Fig. 2). However, when present, the P2 following hand or foot stimulation remained stable in amplitude (hands (2.3 ± 0.5 μV): *P* = 0.864; feet (2.6 ± 0.5 μV): *P* = 0.674) and topography (DISS vs. CGA, hands: *P* = 0.122; feet: *P* = 0.378, Figs 4 and 5). This was maximal at the midline for both hands (94.3%) and feet (90.9%) independently of the CGA of the infants (hands: *P* = 0.116; feet: *P* = 0.313, Figs 4 and 5) with a medial pericentral source (Figs 6 and 7).

N3 was recorded following 84.5% of the hand stimulations independently of CGA (*P* = 0.115, Fig. 2), with a stable amplitude (−2.3 ± 0.5 μV: *P* = 0.835), but its topography changed with age (DISS vs. CGA, *P* = 0.005, Fig. 4) with a shift from contralateral (100% before 36 weeks CGA with a contralateral pericentral source, Fig. 6) to midline (*P* = 0.004, no reliable source localization, Fig. 4). N3 was not recorded following any of the foot stimulations before 36 weeks CGA and increased in occurrence with CGA (*P* = 0.001, Fig. 3). When present, N3 following foot stimulation remained stable in amplitude (−2.5 ± 0.6 μV: *P* = 0.614) and topography (DISS vs. CGA, *P* = 0.852, Fig. 5), which was maximal at the midline 92.3% of the times independently of the CGA (*P* = 0.754, Fig. 5) with a medial pericentral source from early-term (Fig. 7).

## Discussion

We mapped the maturation of the hierarchical processing of tactile inputs in the developing human brain from the late pre-term (34–36 weeks CGA) to full-term age (up to 42 weeks CGA) using SEPs. We found that mechanical stimulation of hands and feet evokes a sequence of 4 deflections representing different levels of this hierarchy: P1, N2, P2, and N3. While the short-latency P1 (lowest processing level) is already developed at 34 weeks CGA, the later potentials (higher processing levels) mature between 34 and 42 weeks CGA. We were also able to localize the source of many of the potentials observed at different ages despite the limited number of electrodes that can be applied to the scalp of a neonate. Here, we frame our results within existing evidence from studies which used other imaging techniques in neonates and adults (e.g., fMRI and magnetoencephalography (MEG)), and animal studies. Nevertheless, the source of the observed potentials cannot be unequivocally attributed to specific cortical areas because of the inherently low spatial resolution of 18 channels EEG recordings, the lack of individual MRI, and co-registration between the functional and anatomical data, and the rapidly changing brain anatomy in this developmental period.

### Short-Latency: P1

P1 can be recorded following median and tibial nerve electrical stimulation from as early as 29 weeks CGA (Hrbek et al. 1973; Gilmore et al. 1987; Karniski et al. 1992; Pike et al. 1997) and, in adults, has been attributed to generators in SI, potentially in BA 3b (Allison et al. 1992; Papadelis et al. 2011), representing the arrival of the peripheral afferent input to the cortex (Papadelis et al. 2012). This indicates that thalamic-SI pathways for both upper and lower limbs are in place, consistent with evidence of maturity of these tracts by 31 weeks according to postmortem (Flower 1985; Volpe 2009) and functional MRI measures (Dall’Orso et al. 2018). Here, we confirm that the same potential can be evoked with mechanical stimulation of hands and feet from 34 weeks CGA and demonstrate that its source is consistent with primary somatosensory representations in SI allowing somatotopically organized information to be available for processing within higher-order brain regions: a pre-requisite of hierarchical somatosensory processing (Thivierge and Marcus 2007). Indeed, animal models confirm that hierarchical propagation of somatosensory-evoked cortical activity depends upon this initial activation (Quairiaux et al. 2011).

### Long-Latency: N2

N2 emerges over the last trimester of gestation and can be evoked with face, hand and foot stimulation from as early as 31–34 weeks CGA (Hrbek et al. 1973; Karniski et al. 1992; Taylor et al. 1996; Pike et al. 1997; Fabrizi et al. 2011; Donadio et al. 2018). At full-term, it is maximal at the vertex and thought to be generated from bilateral post-central gyrus (Karniski et al. 1992). Here, we show that even if present for hands and feet at 34 weeks CGA, the N2 electric field changes with development as the main negative peak shifts from an early purely unilateral distribution with a contralateral pericentral source to more midline at full-term.

The respective contralateral and midline topography and source position of N2 to hand and foot stimulation in the youngest group are consistent with the somatotopic representation of the body in SI. The observed shift in topography to hand stimulation could then represent an increase in the involvement of the ipsilateral homologous region in the generation of this potential, as the electric field from both hemispheres summate at the midline, while canceling out elsewhere (Scherg and Von Cramon 1985). Such a developmental change would not be observed following foot stimulation because of the proximity of the contra and ipsilateral representation of this body part in SI. However, this could not be confirmed by our source localization analysis possibly due to the high variability of the localization for this potential leading to a non-reliable solution.

While P1 is likely generated by BA 3 in SI which does not receive callosal connections even in adulthood and therefore remains a lateralised potential throughout life (Shanks et al. 1985), the latency and largely symmetrical topography of N2 at full-term would be consistent with a source beyond BA 3, such as BA 2. BA 2 is still within SI but receives connections from the homologous region of the other hemisphere allowing a first bilateral integration of somatosensory information (Keysers et al. 2010). In line with this idea, functional and structural studies in humans and animal models have highlighted the emergence of inter-hemispheric communication over the last trimester of gestation or its equivalent (Seggie and Berry 1972; Erberich et al. 2006; Yang et al. 2009; Quairiaux et al. 2011; McVea et al. 2012; Allievi et al. 2016; Kozberg et al. 2016). In this context, the decreasing amplitude of the N2 observed here is concordant with increasing inter-hemispheric inhibition mediated by the corpus callosum (Marcano-Reik et al. 2010).

Unlike the P1 which has a consistent topography across development, the N2 reflects a clear change between the pre-term and late-term period at this second level of the somatosensory hierarchy. The maturation of this processing level, which potentially allows the discrimination of bilateral tactile stimuli, occurs over the period at which birth into the extra-uterine environment is due (37–40 weeks). As this is the age at which feeding begins, during which infants typically grasp the breast with their palms (Colson et al. 2008), the ability to integrate bilateral somatosensory input would be advantageous to this end.

### Long-Latency: P2

P2 emerges over the last trimester of gestation, can be evoked with face, hand, and foot stimulation and, at full-term, is maximal at the vertex (Hrbek et al. 1973; Fabrizi et al. 2011; Nevalainen et al. 2015; Donadio et al. 2018). Here, we show that P2 is already established for the upper limbs at 34 weeks CGA, but is still emerging for the lower limbs at this age, providing the first evidence of a rostro-caudal developmental gradient of somatosensory functions in humans. Moreover, considering that P2 is still emerging while N2 is already established, this indicates a hierarchical developmental gradient, where higher levels of the somatosensory processing stream develop later. This is in line with rodent models which demonstrate later components emerging within the somatosensory response from P16 (Quairiaux et al. 2011).

The earlier development of the P2 to hand stimulation, compared with foot stimulation, could arise from faster maturation of cortico-cortical pathways from hand areas of SI to other associative areas. In line with this, in rat pups SI cortex is only clearly separated into columns, thus facilitating efficient outputs, for their most important body surfaces (vibrissae and forelimbs) (Armstrong-James 1975). Equivalent preferential development of upper limb somatosensory circuits is indicated in humans by the fact that, perinatally, only the pericentral gyri corresponding to hand representations are myelinated (Barkovich et al. 1988). Fine hand function is advantageous as soon as infants enter the extra-uterine environment, e.g., for breast-feeding as described above. Therefore, early maturation of upper versus lower limb somatosensory pathways may confer this advantage.

The vertex topography of the P2 with a stable midline pericentral source for both hands and feet is consistent with a medial structure with no somatotopic arrangement such as the Supplementary Motor Area (SMA), which is involved in motor preparation (Mima et al. 1999; Cunnington et al. 2003) and is strongly integrated into the somatosensory response in infants from 34 weeks (Allievi et al. 2016), adults (Burton et al. 1993; Ruben et al. 2001) and non-human primates (Wong et al. 1978). In adults, SMA can respond to contralateral and ipsilateral somatosensory stimulation and is therefore capable of bilateral activation following unilateral stimulation. In infants, inter-hemispheric functional connections between the right and left SMAs are in place from the late pre-term period (Barkovich et al. 1988; Smyser et al. 2010), facilitated by the accelerated development of the callosal tracts linking the frontal lobes from 33 weeks (Rakic and Yakovlev 1968). Scalp topography and source localization would not reflect these developmental changes because of the proximity to the midline of the SMAs.

Moreover, activation of SMA is likely to occur in parallel with activation of the secondary somatosensory cortex (SII) and posterior parietal cortex (BA 5 and 7) which subserve tactile object recognition, internal body image, and integrate somatosensory with visual information to facilitate eye-hand coordination (Kandel et al. 2000; Dijkerman and Haan 2007; Keysers et al. 2010; Leib et al. 2016). SII opercular and posterior parietal cortices are structurally interconnected and, in adults, are simultaneously active at 50–140 ms after a somatosensory stimulus (Allison et al. 1989; Mauguière et al. 1997; Keysers et al. 2010). In line with this, MEG experiments—which are most sensitive to opercular generators—have also identified activation of SII cortex at a comparable latency to our P2 following taps to the hand of infants ≥38 weeks CGA (Nevalainen et al. 2015). Taken together, the P2 potential could be associated with activation of a SMA source, which is likely to occur together with activation of SII and posterior parietal cortex supporting parallel processing streams by full-term age.

### Long-Latency: N3

The N3 potential is scarcely reported in the literature. Here, we show that in pre-term infants the N3 is evoked by hand but not foot stimulation, reinforcing the idea that upper limb pathways within the higher levels of the somatosensory hierarchy mature earlier. However, this potential follows a similar developmental topographic shift to N2 which suggests an increasingly bilateral cortical generator and therefore an initially not fully developed hand response too. Taken together, these findings indicate that this potential is the latest to mature which is consistent with it representing the very highest level of somatosensory processing for newborn infants. The contralateral topography and source position to hand stimulation and midline topography to foot stimulation in the youngest group are consistent with the somatotopic representation of the body in SI and the similar topographic shift to N2 following hand stimulation suggests a shared or nearby generator. Forward projections to associative areas from SI are reciprocated by backward projection (Friedman 1983; Cauller et al. 1998), so the N3 recorded here could represent a successive re-activation of the neuronal population that earlier generated the N2. This would explain its late-maturation, as feedback projections develop after feedforward ones (Berezovskii et al. 2011). Top-down re-activation is hypothesized to bind together parallel streams of sensory feature analysis, and predict future sensory inputs (Cauller et al. 1998; Berezovskii et al. 2011).

### Summary

There is a dynamic evolution in hierarchical somatosensory processing across the late pre-term and perinatal period. 4 separate potentials comprise the neonatal somatosensory response: P1, N2, P2, and N3. The initial P1 potential is consistent with an elementary SI generator while the subsequent N2 potential is concordant with a higher-level generator such as BA 2. Both potentials are present from 34 weeks but, while the P1 remains stable, the changing topography of the N2 suggests increased involvement of the ipsilateral hemisphere in somatosensory processing. Meanwhile, the P2 and N3 have a unique developmental profile which indicates a gradient in the maturation of cortical somatosensory processing starting from the upper before the lower limbs.

**Figure 8.**
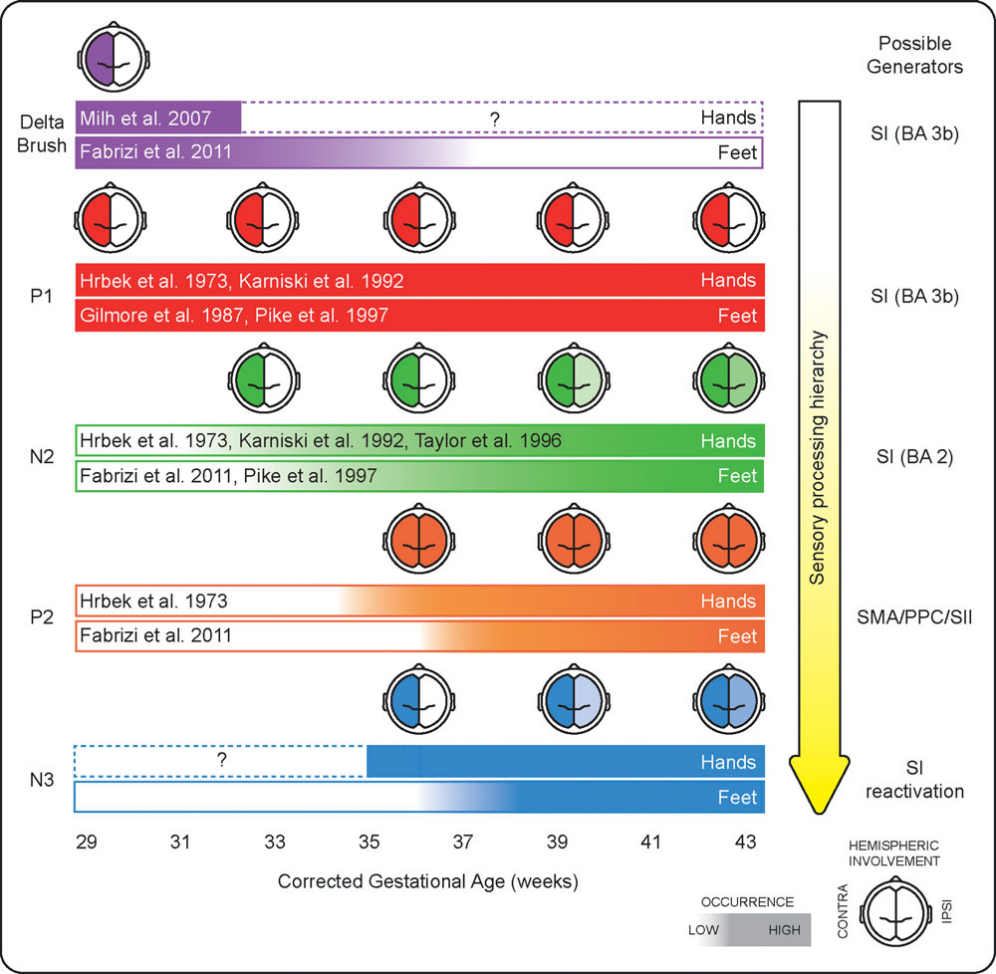
Summary of the current evidence (including the present study) about the emergence and hemispheric involvement for the SEPs representing the different stages of the somatosensory processing hierarchy for hands and feet stimulation. On the right-hand side, possible generators of each potential are summarized (SI, primary somatosensory cortex; SMA, supplementary motor area; PPC, posterior parietal cortex; SII, secondary somatosensory cortex).

The late potentials following stimulation of the body surface could represent a neuronal marker for higher-order somatosensory processing in late pre-term and full-term infants (Fig. 8). Investigating how experience-dependent processes shape the development of this hierarchical somatosensory system will be an important next step in understanding why some pre-term infants develop sensorimotor difficulties.

## Supplementary Material

Supplementary Data
