# Supplementary material for: The Emergence of Hierarchical Somatosensory Processing in Late Prematurity
Source: Cereb Cortex. 2019 Mar 7;29(5):2245–60. doi: 10.1093/cercor/bhz030 (PMC6458926; doi:10.1093/cercor/bhz030)
Supplement: Supplementary Data [file bhz030_supplementary_materials.zip › bhz030_Supplementary_material_final.docx]

**Supplementary Material**


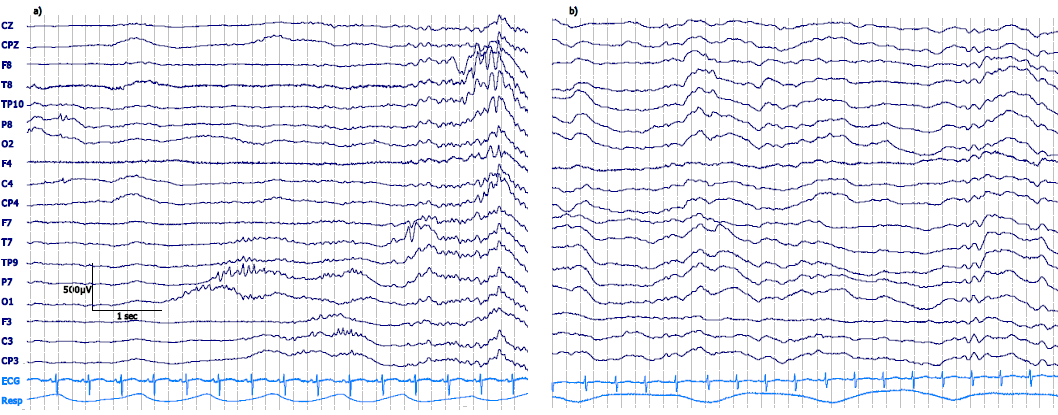


**Supplementary Figure 1**. Resting EEG during quiet sleep in infants of (a) CGA 35+0 and (b) CGA 42+5 weeks. Note the increased continuity of the EEG in the older infant. Data are displayed referred to Fz (acquisition montage) and low-pass filtered at 70 Hz.


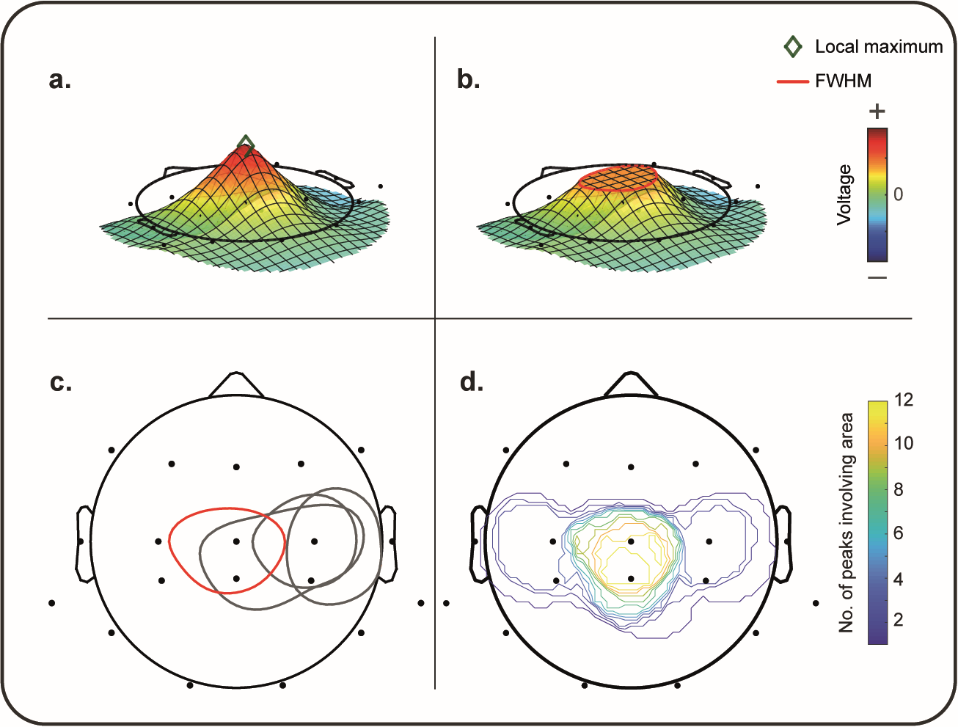


**Supplementary Figure 2.** Pipeline for the definition of spatial region of interest (ROI) for individual peak detection. (a) Identification of local spatial maximum/minimum at the latencies of one of the grand average SEPs (in this example P2 following left hand stimulation); (b) mark equipotential line at half maximum (i.e. 2D Full-Width at Half-Maximum, FWHM); (c) repeat (a-b) for every SEP following the stimulation of one limb (in this example left hand stimulation, in red is the same FWHM contour shown in (b)); (d) repeat (c) for every limb. The spatial ROI for individual peak identification is the union of the ROIs defined for every SEP following stimulation of every limb (Figure 1).


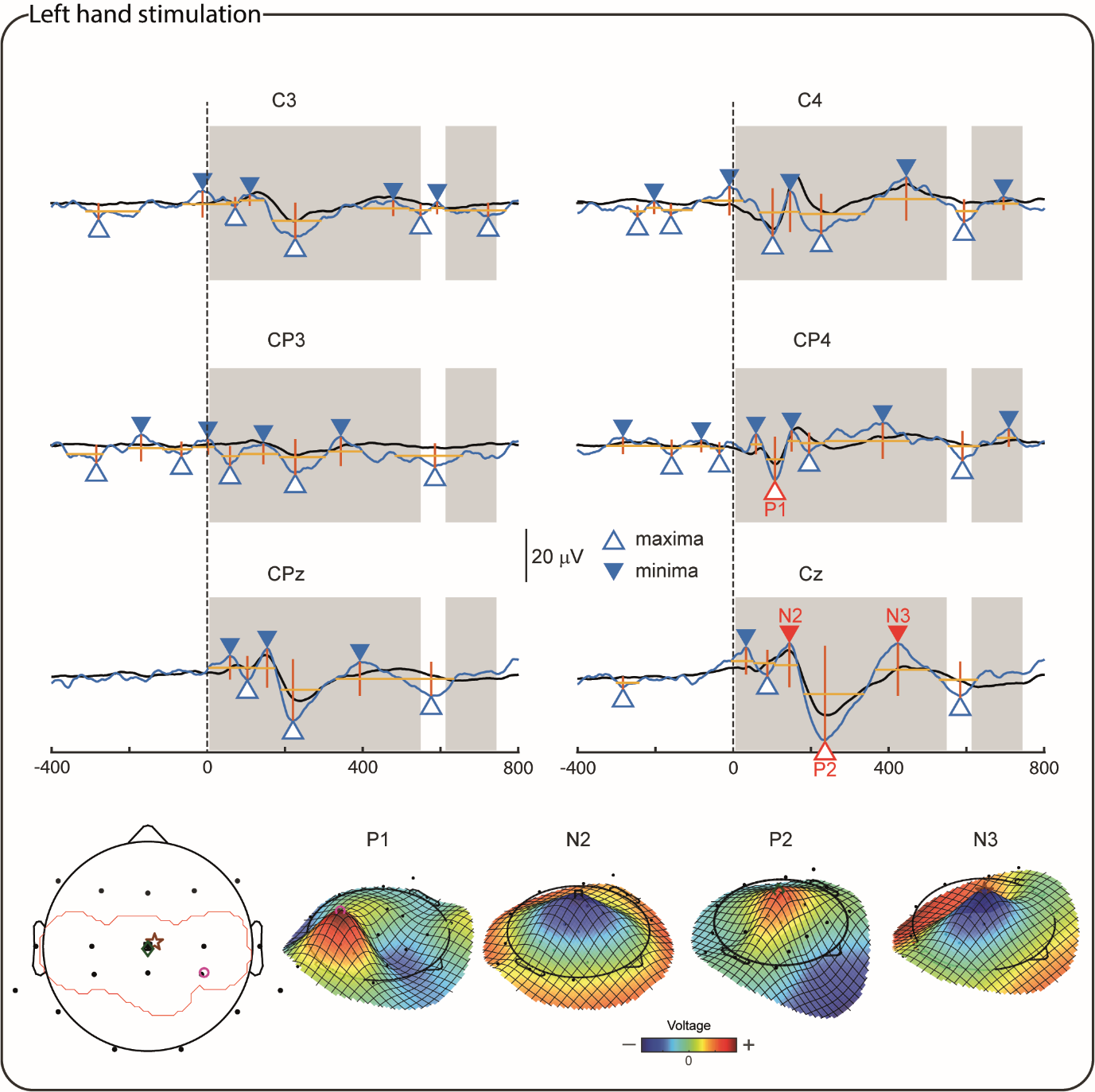


**Supplementary Figure 3.** Example of identification of individual SEPs in the response to left hand stimulation of a 41 weeks CGA subject. Peaks potentially representing SEPs were first identified as local maxima/minima in the time domain from the recording at the pericentral electrodes (top panel). These were data samples that were larger (empty triangles) or smaller (full triangles) than their two neighbouring samples, had a prominence of more than 2 μV, width at half-prominence of more than 14 ms and occurred within the temporal ROI (shaded time interval). If more data points satisfied these criteria the latency of that closest to the grand average SEP was selected (red triangles). If a peak potentially representing an SEP was present in the time domain, its topography was assessed against the spatial ROI derived from the grand averages (bottom panel). Spatial maxima/minima at the latencies of the selected peaks were identified. If the spatial maxima/minima fell within the boundary of the spatial ROI the SEP was finally considered present.

**Supplementary Video 1.** Example of tactile stimulation of the left hand in a 37+2 weeks CGA infant. Solid vertical purple lines indicate the occurrence of a tap. The trace of the electrode overlying the primary cortical representation of the stimulated hand (C4) is emphasised (amplitude x2). Data are displayed referred to Fz (acquisition montage) and band-pass filtered 1.5-40 Hz.

**Supplementary Video 2.** Example of tactile stimulation of the right foot in a 37+2 weeks CGA infant. Solid vertical purple lines indicate the occurrence of a tap. The trace of the electrode overlying the primary cortical representation of the stimulated foot (Cz) is emphasised (amplitude x2). Data are displayed referred to Fz (acquisition montage) and band-pass filtered 1.5-40 Hz.
